# Supplementary material for: Analytical Perspectives in the Study of Polyvalent Interactions of Free and Surface-Bound Oligonucleotides and Their Implications in Affinity Biosensing
Source: Int J Mol Sci. 2022 Dec 22;24(1):175. doi: 10.3390/ijms24010175 (PMC9820729; doi:10.3390/ijms24010175)
Supplement: Supplementary file 1 [file ijms-24-00175-s001.zip › ijms-2103977-supplementary.pdf]

Supplementary Information

Analytical Perspectives in the Study of Polyvalent Interactions of Free and Surface-Bound Oligonucleotides and Their Implications in Affinity Biosensing

Laura-Elena Gliga [1,†], Bogdan-Cezar Iacob [1,†], Sanda-Nastasia Moldovean [2], David A. Spivak [3], Andreea Elena Bodoki [4], Ede Bodoki [1,*] and Radu Oprean [1]

[1] Analytical Chemistry Department, "Iuliu Hațieganu" University of Medicine and Pharmacy, 4, Louis Pasteur St., 400349 Cluj-Napoca, Romania
[2] Faculty of Physics, Babeș-Bolyai University, 1, Kogălniceanu St., 400084 Cluj-Napoca, Romania
[3] Department of Chemistry, Louisiana State University, Baton Rouge, LA 70803, USA
[4] Inorganic Chemistry Department, "Iuliu Hațieganu" University of Medicine and Pharmacy, 12, Ion Creangă St., 400010 Cluj-Napoca, Romania
* Correspondence: bodokie@umfcluj.ro
† These authors contributed equally to this work.

[1] **Equal contributions.**

Supplementary Figure S1

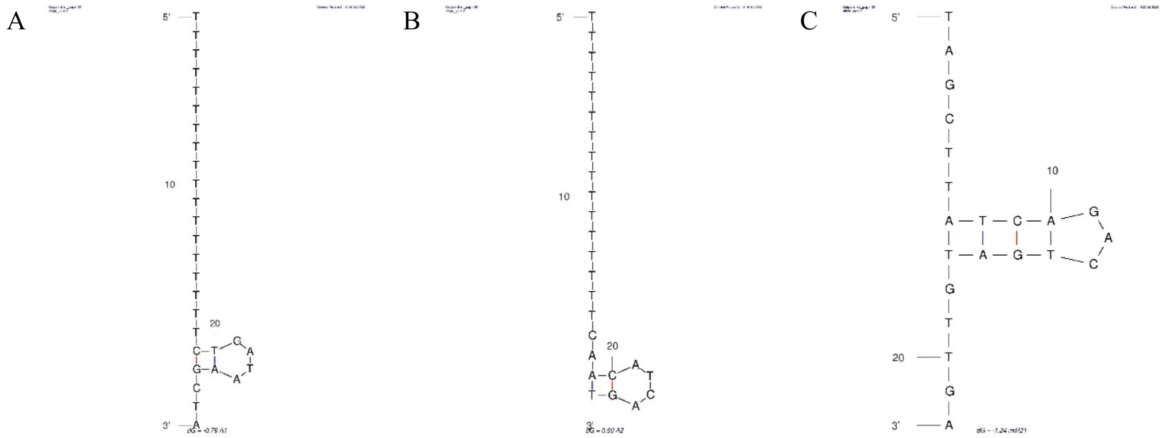

Figure S1. Predicted secondary structures of (A) A1, (B) A2 and (C) miR21 oligonucleotides generated from the UNAFold Web server [1]

Supplementary Figure S2

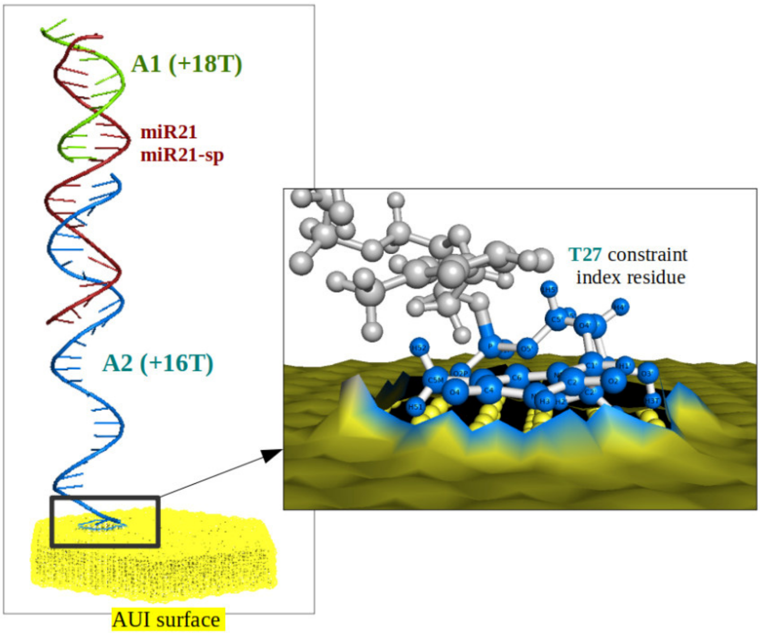

Figure S2 The input 3D structure of the interacting systems

Supplementary Figure S3

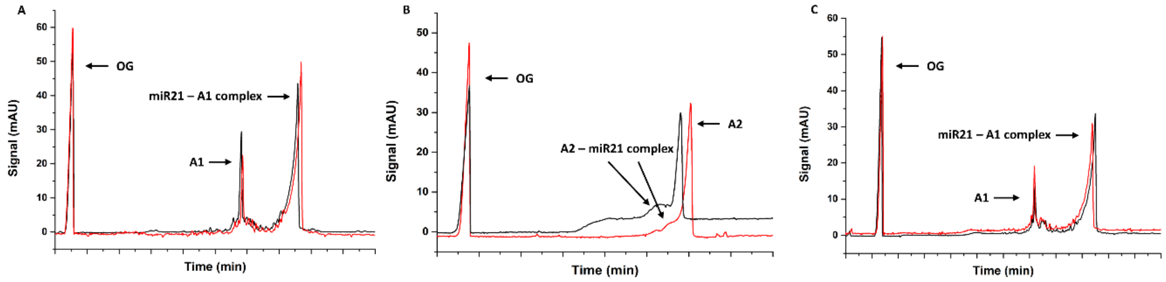

Figure S3 (A) miR21 (1.11 µM) – A1 (2.22 µM) interaction with (red) or without (black) thermal denaturation of aptamers, (B) miR21 (1.11 µM) – A2 (2.22 µM) interaction with (red) or without (black) thermal denaturation of aptamers, (C) miR21 (1.11 µM) – A1 (2.22 µM) interaction (C) in the presence (red) or in the absance (black) of Mg(II) ions

Supplementary Figure S4

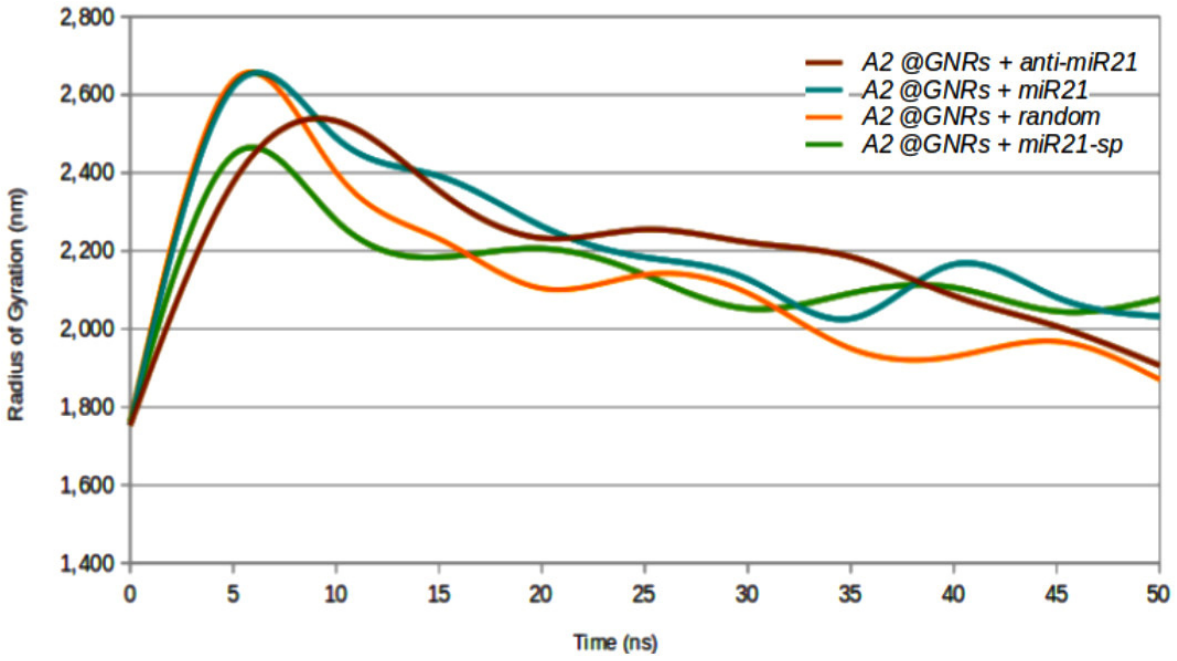

Figure S4 Radius of gyration profiles for the binary interacting systems with A2@GNRs

Supplementary Figure S5

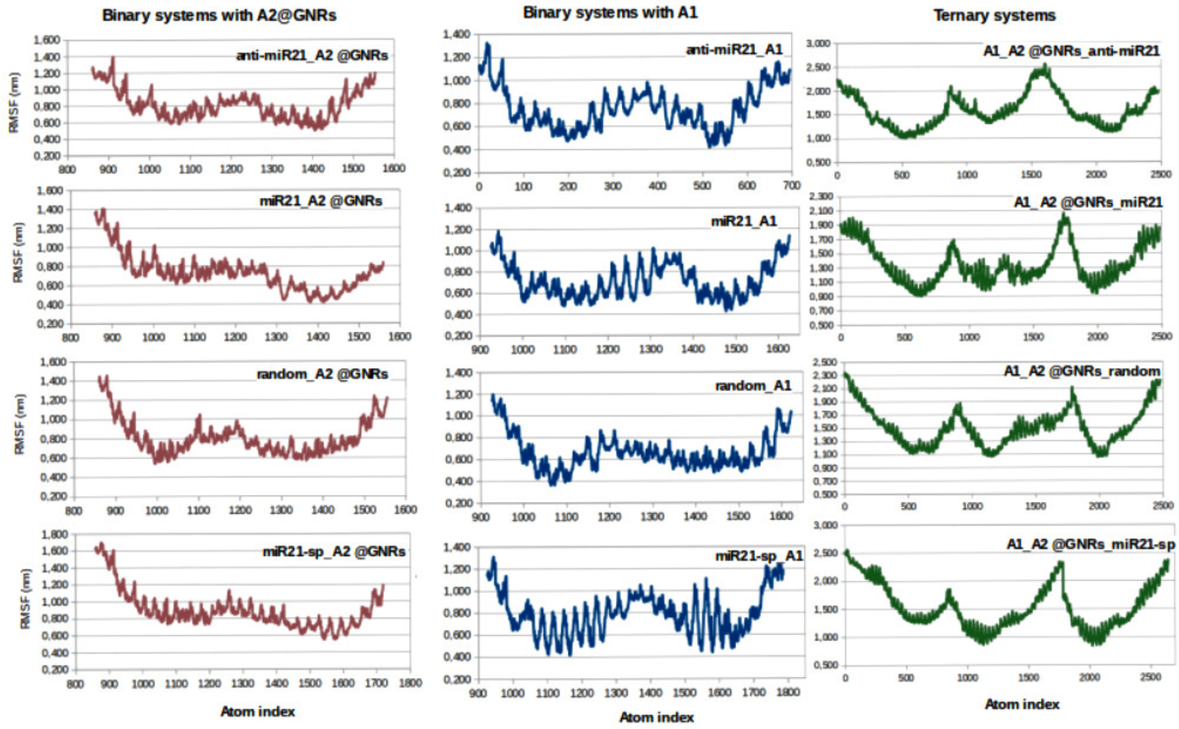

Figure S5 Atomic RMS fluctuations for all interacting complexes.

Supplementary Figure S6

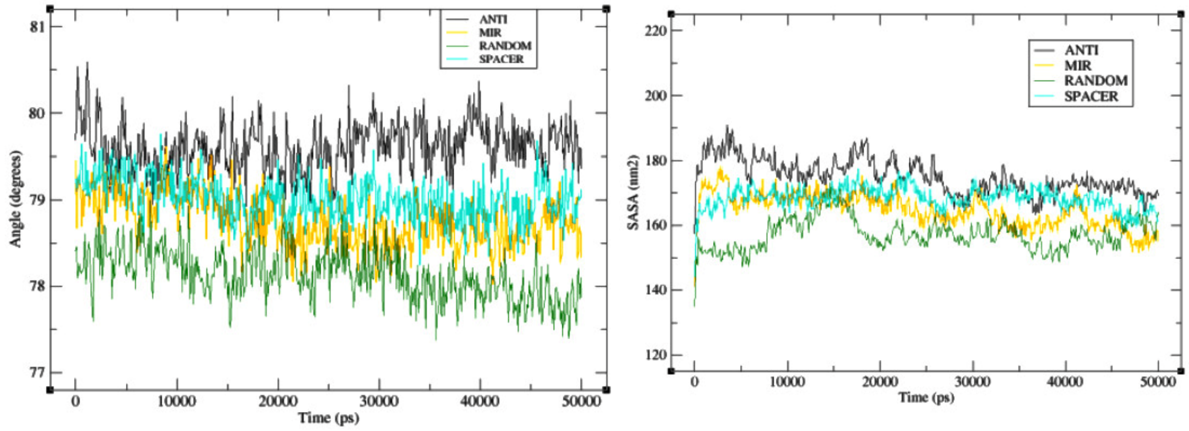

Figure S6 Average angles (left) and solvent exposure surface area (right) measurements for the ternary interacting systems.

Supplementary Figure S7

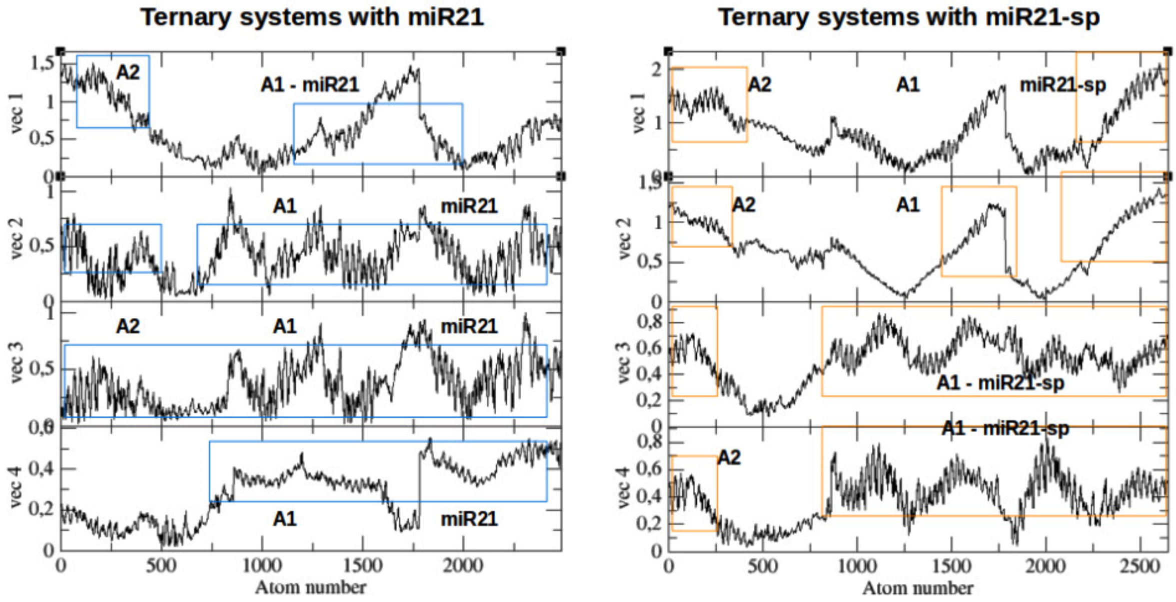

Figure S7 RMSF extracted from PCA analysis of the first 4 vectors for A1 – A2 – APT interacting systems

[1] N.R. Markham, M. Zuker, UNAFold BT - Bioinformatics: Structure, Function and Applications, in: J.M. Keith (Ed.), Humana Press, Totowa, NJ, 2008: pp. 3–31. https://doi.org/10.1007/978-1-60327-429-6_1.
